# MRI Risk Stratification for Tumor Relapse in Rectal Cancer Achieving Pathological Complete Remission after Neoadjuvant Chemoradiation Therapy and Curative Resection

**DOI:** 10.1371/journal.pone.0146235

**Published:** 2016-01-05

**Authors:** Honsoul Kim, Sungmin Myoung, Woong Sub Koom, Nam Kyu Kim, Myeong-Jin Kim, Joong Bae Ahn, Hyuk Hur, Joon Seok Lim

**Affiliations:** 1 Department of Radiology, Research Institute of Radiological Science, Yonsei University College of Medicine, Seoul, 120–752, Republic of Korea; 2 Department of Medical Information, Jungwon University, Goesan, 367–805, Republic of Korea; 3 Department of Radiation Oncology, Yonsei University College of Medicine, Seoul, 120–752, Republic of Korea; 4 Department of Surgery, Division of Colon and Rectal Surgery, Yonsei University College of Medicine, Seoul, 120–752, Republic of Korea; 5 Department of Internal Medicine, Yonsei University College of Medicine, Seoul, 120–752, Republic of Korea; H. Lee Moffitt Cancer Center & Research Institute, UNITED STATES

## Abstract

**Purpose:**

Rectal cancer patients achieving pCR are known to have an excellent prognosis, yet no widely accepted consensus on risk stratification and post-operative management (*e*.*g*., adjuvant therapy) has been established. This study aimed to identify magnetic resonance imaging (MRI) high-risk factors for tumor relapse in pathological complete remission (pCR) achieved by rectal cancer patients who have undergone neoadjuvant concurrent chemoradiation therapy (CRT) and curative resection.

**Materials and Methods:**

We analyzed 88 (male/female = 55/33, median age, 59.5 years [range 34–78]) pCR-proven rectal cancer patients who had undergone pre-CRT MRI, CRT, post-CRT MRI and curative surgery between July 2005 and December 2012. Patients were observed for post-operative tumor relapse. We analyzed the pre/post-CRT MRIs for parameters including mrT stage, mesorectal fascia (mrMRF) status, tumor volume, tumor regression grade (mrTRG), nodal status (mrN), and extramural vessel invasion (mrEMVI). We performed univariate analysis and Kaplan-Meier survival analysis.

**Results:**

Post-operative tumor relapse occurred in seven patients (8.0%, n = 7/88) between 5.7 and 50.7 (median 16.8) months. No significant relevance was observed between tumor volume, volume reduction rate, mrTRG, mrT, or mrN status. Meanwhile, positive mrMRF (P_pre-CRT_ = 0.018, P_pre/post-CRT_ = 0.006) and mrEMVI (P_pre-CRT_ = 0.026, P_pre-/post-CRT_ = 0.008) were associated with higher incidence of post-operative tumor relapse. Kaplan-Meier survival analysis revealed a higher risk of tumor relapse in patients with positive mrMRF (P_pre-CRT_ = 0.029, P_pre-/post-CRT_ = 0.009) or mrEMVI (P_pre-CRT_ = 0.024, P_pre-/post-CRT_ = 0.003).

**Conclusion:**

Positive mrMRF and mrEMVI status was associated with a higher risk of post-operative tumor relapse of pCR achieved by rectal cancer patients, and therefore, can be applied for risk stratification and to individualize treatment plans.

## Introduction

Neoadjuvant concurrent chemoradiation therapy (CRT) has been increasingly used to treat locally advanced rectal cancer. It has been proven to reduce local tumor recurrence and toxicity when compared with post-operative chemoradiation therapy [1]. Moreover, approximately 10–30% of rectal cancer patients achieve pathological complete remission (pCR) after CRT [2–4]. These patients are known to carry a very low risk of lymph node (LN) metastasis and to experience excellent overall and disease-free survival [5–7]. Therefore, if a rectal cancer patient who has undergone CRT and subsequent surgery is documented as pCR, then a preferable prognosis can be anticipated.

With the widespread use of CRT in rectal cancer, we now encounter increasing numbers of patients who reach pCR. However, this encouraging condition of absent residual viable tumor cells upon histopathological analysis has raised several issues.

One unsolved controversy is how to manage pCR achieved rectal cancer patients after standard treatment. Currently, CRT followed by radical surgery is the standard treatment of locally advanced rectal cancer and ensures excellent local control of pCR patients. The prognosis of pCR achieved patients are expected to be excellent, therefore it is not clear whether these patients should be managed same as other patients who do not achieve pCR. Collectively, we envision that risk stratification of pCR patients who already completed standard CRT and curative surgery is necessary.

MRI is an established imaging tool, which is widely used for local staging of rectal cancer. We hypothesized that certain MRI features may be associated with a higher risk of tumor relapse in pCR rectal cancer patients after surgery, which could be applied for risk stratification, useful towards individualizing treatment strategies, as well as predicting prognosis.

The purpose of this study was to identify high-risk MRI features associated with tumor relapse in pCR-achieved rectal cancer patients who had undergone standard CRT and curative surgery.

## Material and Methods

### Study population and identification of tumor-recurred patients

This retrospective study was approved by the institutional review board of Severance Hospital, and a waiver for informed consent was obtained (Approval number: 4-2014-0177). All the data were analyzed anonymously. We screened 93 consecutive pCR (Mandard grade 1) rectal cancer patients who had undergone an elective resection after CRT between July 2005 and December 2012. We defined the pCR state as having no residual viable tumor cells in the primary rectal mass irrespective of lymph node involvement. Those patients lacking pre- and/or post-CRT rectal MRIs (n = 5) were excluded. No patients had evidence of synchronous distant metastasis at the pre-operative period. Ultimately, our final study population consisted of 88 pCR patients (male/female = 55/33, and median age, 59.5 years [range 34–78]).

Patient and tumor characteristics, including the type of treatment, serum CEA, and pathologic results were noted. Medical records and reports of post-operative surveillance imaging studies were reviewed until December 2014 to identify tumor relapse and/or mortality events. The median follow-up period, calculated from the date of surgery, was 49.0 months (range, 17.7 to 106.7 months). The relapse-free period (RFP) was defined as the time period between the date of the surgery and the date when tumor recurrence was first diagnosed.

### Patient treatment

All patients received CRT. The principle of deciding to proceed with pre-CRT was based on 1) a clinical MR stage of T3/T4 cancer irrespective of the N stage or any T with positive N status, or 2) any lower rectal cancer located near the sphincter complex, and 3) no identifiable distant metastasis detectable by other imaging modalities. CRT was performed as previously described [8]. Radiation therapy was delivered at an energy level of 6 MV/10 MV. A total dose of 45 Gy was irradiated in 25 fractions to the pelvis over the course of five weeks (1.8 Gy/day, five days/week). A subsequent 5.4 Gy boost targeting the primary tumor was delivered. The regimen of concurrent chemotherapy included 5-fluorouracil and leucovorin (n = 60), capecitabine (n = 6), and S-1 plus irinotecan (n = 22). Patients underwent surgery between six and eight weeks following the completion of CRT. The standard method of surgery was total mesorectal excision, and curative resection was performed on all patients without residual cancer. Surgical resection was classified as low anterior resection, ultra-low anterior resection, or abdominoperineal resection. After surgery, 65 patients received adjuvant chemotherapy either with the FOLFOX protocol (n = 59), capecitabine (n = 5), or the FOLFIRI protocol with bevacizumab (n = 1), while 23 patients did not receive adjuvant chemotherapy (Table 1).

**Table 1:** Patient characteristics.

Variables	No. Patients	%
**Age (years)**		
Range 34–78 (median 59.5)		
**Sex**		
Male	55	(63)
Female	33	(38)
**Tumor level (MR classification of the distal margin)**		
Upper	1	(1)
Middle	26	(30)
Lower (including anal canal)	61	(69)
**Histologic grade (biopsy)**		
Well-differentiated	21	(24)
Moderately differentiated	61	(69)
Poorly differentiated	3	(3)
Mucinous	3	(3)
**Pathologic LN metastasis**	4	(5)
**Pre-CRT protocol**		
5-fluorouracil+leucovorin	60	(68)
Capecitabine	6	(7)
Irinotecan/TS-1	22	(25)
**Adjuvant chemotherapy**		
None	23	(26)
FOLFOX protocol	59	(67)
Capecitabine	5	(6)
FOLFIRI protocol + Avastin	1	(1)
**Type of resection**		
Low anterior resection	53	(60)
Ultra-low anterior resection with colo-anal anastomosis	31	(35)
Abdominoperineal resection	4	(5)

CEA, carcinoembryonic antigen; CRT, neoadjuvant concurrent chemoradiotherapy; LN, lymph node; MR, magnetic resonance.

### Image acquisition and analysis

Post-CRT MRIs were obtained 3–5 weeks before surgery. MR imaging was performed as previously described [9, 10] with either a 1.5T scanner (Achieva; Philips Medical Systems, Best, The Netherlands) or 3.0T scanner (Magnetom Tim Trio; Siemens Medical Solutions, Erlangen, Germany). T2-weighted MR images were obtained in axial, sagittal, oblique axial, and coronal orientations using a respiratory-triggered echo train spin echo sequence.

All MR images were analyzed on a picture-archiving and communicating system workstation (Centricity, GE Healthcare, Milwaukee, WI, USA). Two gastrointestinal radiologists (H.K. and J.S.L., with four years and 12 years of experience in abdominal imaging, respectively) specializing in rectal cancer evaluation, retrospectively reviewed the MR images on consensus. The reviewers were aware that the patients achieved a pCR state, but were blinded to all other clinical information.

The pre-CRT MRI T2-weighted sagittal image was used to classify the tumor’s distal margin level (upper rectum, n = 1; middle rectum, n = 26; lower rectum & anal canal, n = 61): Three imaginary lines, each connecting the center of the symphysis pubis and 1) sacral promontory, 2) peritoneal reflection, and 3) intervertebral junction between the 5^th^ sacral bone and the coccyx were drawn to divide the rectum into three compartments (S1 Fig).

To measure the tumor volume, the T2-weighted oblique axial images were archived in Digital Imaging and Communications in Medicine (DICOM) format, and stored on a General Electric Advantage workstation version 4 (GE Healthcare, Waukesha, WI, USA). The region of interest (ROI) was drawn along the margin of the rectal mass on each slice, and the area of the ROI obtained from each image was added to calculate the total mass volume. The volume reduction rate was calculated according to the following equation: volume reduction rate (%) = (Volume_pre-CRT_−Volume_post-CRT_)/Volume_pre-CRT_ ☓ 100.

The MRI tumor regression grade (mrTRG) was assigned into one of the two categories (mrTRG_1/2_ vs. mrTRG_3/4/5_) to simplify statistical analysis based on the comparison of pre-/post-CRT MRIs. For mrTRG_1/2_, the residual tumor is absent (mrTRG_1_) or only a small residual tumor with a predominant fibrotic low-signal intensity remains (mrTRG_2_). For mrTRG_3/4/5_, all other lesions are included that do not meet the criteria of mrTRG 1 or 2 (modified from [11]).

The MRI T (mrT) stage was classified as a low mrT stage group (mrT1/2: tumor is confined within rectal muscularis propria and mrT3_<5mm_: tumor extent is <5 mm beyond the muscularis propria) or as a high mrT stage group (mrT3_≥5mm_: tumor extent ≥5 mm beyond muscularis propria and mrT4: invasion of other organs) [11]. A patient was considered to be an mrT down-staged case if graded as mrT3_≥5mm_ on pre-CRT but mrT3_<5mm_ on post-CRT MRI.

The MRI mesorectal fascia (mrMRF) was defined as a T2 dark signal intensity linear structure encasing the rectum and perirectal fat. The mrMRF status was graded according to the shortest distance from the tumor outermost margin to the adjacent MRF and labeled as negative (≥2 mm) or positive (<2 mm) [12–14]. If a patient’s MRF status was initially graded as positive on pre-CRT MRI, but on the post-CRT MRI the mass decreased and a fat pad thicker than 2mm appeared between the residual mass (with or without fine speculation) and the MRF, then the post-CRT MRI MRF status was graded as negative [15].

The MRI extramural vessel invasion (mrEMVI) status was graded as negative or positive. EMVI was considered positive when a gross vessel adjacent to the tumor presented with an irregular contour showing either nodular expansion or contained intermediate signal intensity [16].

The MRI regional LN (mrN) status was assigned into one of two groups (negative vs. positive for metastasis). The nodal status was considered negative if the largest LN short diameter was < 6 mm and the LN had an irregular border or mixed signal intensity was absent. Positive LNs were defined as LNs with a short diameter ≥ 6 mm and/or a LN presenting with an irregular border and/or mixed SI [17].

### Statistical analysis

Statistical analysis was done using the SPSS version 20.0 (IBM Corp., Armonk, NY, USA) software package. Fisher’s exact test was used to compare age (≤60, >60), sex, CRT protocol, adjuvant chemotherapy protocol, tumor level, mrTRG, mrT stage, mrMRF status, mrEMVI status, and mrN status. Independent t-test was conducted to compare CEA levels, tumor volume, and volume reduction rate between the tumor-relapsed group and the non-relapsed group.

Survival analysis using the Kaplan-Meier method was performed to calculate the estimated probability of the overall tumor relapse rate and overall survival rate. Time to tumor relapse was calculated from the date of surgery to the date when the tumor relapse was first discovered. Log-rank method was used to compare different data sets. Data were censored at the time of the last follow-up. Because of the small number of tumor recurrences, we did not perform multivariate analysis. Statistical significance was accepted for differences with *P* values of less than 0.05.

## Results

**Fig 1:**
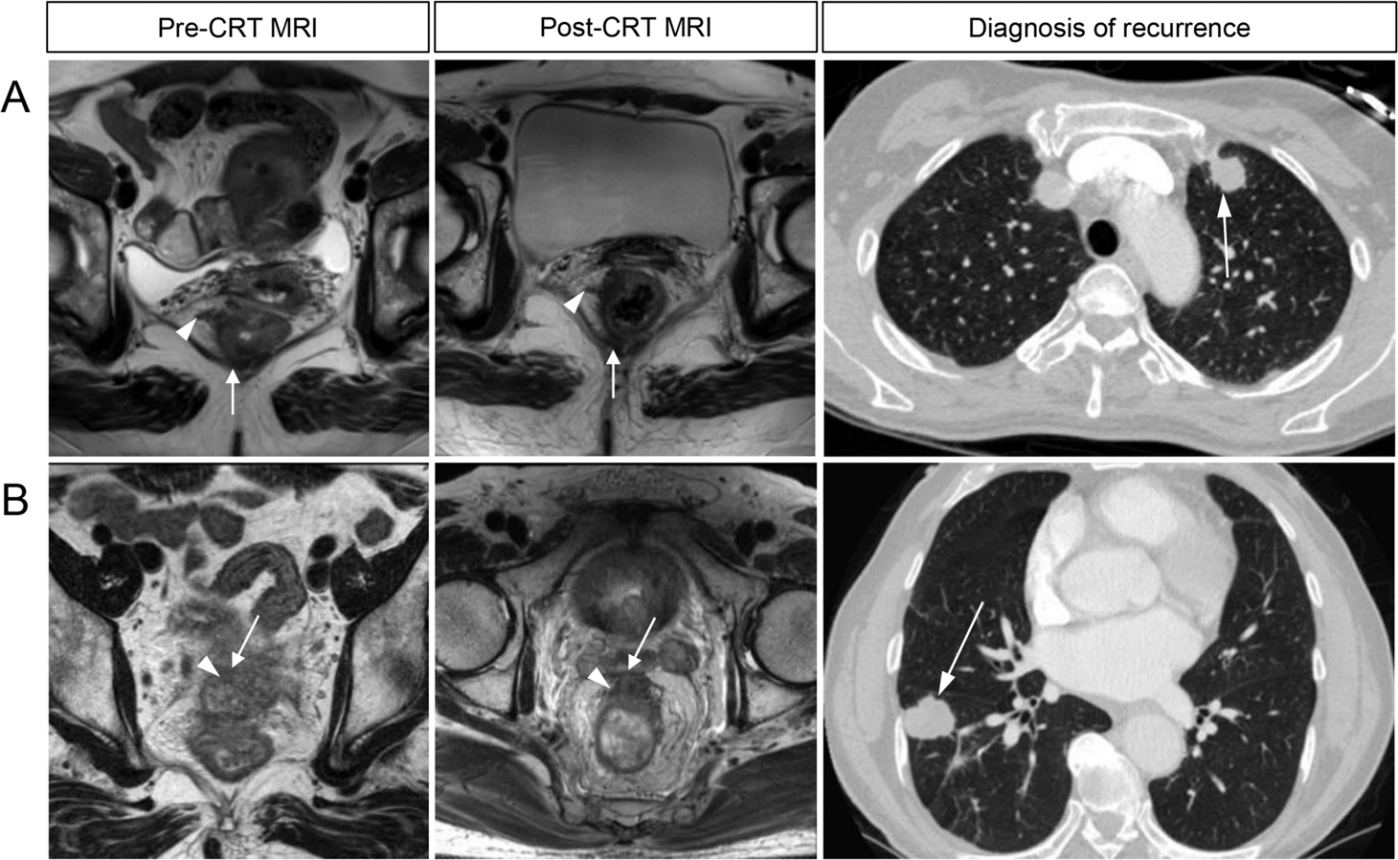
Representative images of a pre-CRT MRI (left column), post-CRT MRI (middle column), and image of a recurred tumor obtained from a pCR patient who later developed tumor recurrence. (A) 50-year-old female with lung metastasis (relapse-free period, 22.9 months), (B) 70-year-old male with lung metastasis (relapse-free period, 37.4 months). Positive MRI mesorectal fascia (short arrow), positive MRI extramural vessel invasion (arrowhead), and a recurred tumor lesion (long arrow) are depicted.

Among the 88 pCR patients, seven tumor relapses occurred (8.0%, n = 7/88) for which the RFP ranged from 5.7 to 50.7 (median 16.8) months. Tumor recurrence occurred at the anastomosis site (n = 1, RFP: 50.7 months), para-aortic space node (n = 1, RFP: 7.3 months), liver (n = 2, RFP: 5.7, 42.2 months), and lung (n = 3, RFP: 10.8, 22.9, 37.4 months) (Fig 1). The level of rectal cancer in these tumor-relapsed patients was as the middle rectum (n = 3) or lower rectum (n = 4). The relapsed tumor lesion was confirmed either by biopsy or elective resection in all seven cases. All seven relapsed patients had received adjuvant chemotherapy before tumor relapse developed. Three deaths occurred among these seven patients due to progression of the relapsed tumor (S1 Table). Meanwhile, the relapsed tumor lesions in the other four patients were successfully controlled by additional surgical interventions and systemic chemotherapy. No mortalities occurred in the non-relapsed group.

The level of the tumor distal margin did not show statistical significance with respect to the tumor relapse (*P* = 0.468). The pre-CRT CEA level between tumor-relapsed patients (16.0±6.1 ng/mL) and non-relapsed patients (4.0±6.0 ng/mL) did not show a statistically significant difference (*P* = 0.471). There were no significant clinical factors associated with tumor relapse in pCR patients (S2 Table).

The tumor volume measured on both the pre- and post-CRT MRIs did not differ between the non-relapse group (pre-CRT: 0.4–144.3, 23.9±24.4 cm^3^; post-CRT: 0–46.2, 7.4±8.9 cm^3^) and the tumor relapse group (pre-CRT: 5.6–81.6: 30.1±24.9 cm^3^; post-CRT: 1.0–32.8: 10.3±11.1 cm^3^), showing no statistically significant difference (*P*_*pre-CRT*_ = 0.516; *P*_post-CRT_ = 0.421). No statistically significant differences were seen in the volume reduction rate (*P* = 0.962) between the tumor non-relapse group (13.3–100: 70.7±19.8%) and the tumor relapse group (50.8–87.2: 70.3±14.0%). The mrTRG assessment did not show a difference in tumor relapse (*P* = 1.0) between mrTRG_1/2_ (n = 26; two tumor relapses) and mrTRG_3/4/5_ (n = 62; five tumor relapses), (S3 Table).

On pre-CRT MRI, the low mrT stage group (n = 46; three tumor recurrences) and the high mrT stage group (n = 42; four tumor recurrences) did not show a significant difference in tumor relapse (*P* = 0.705). Comparison of pre-/post-CRT MR revealed that down staging of the T stage occurred in 14 patients; therefore, the low mrT stage was re-grouped to include 60 patients (46 persistently low mrT stage and 14 down-staged patients). However, no statistically significant difference in terms of tumor recurrence was observed between these two groups (tumor relapse/low mrT vs. persistently high mrT: n = 4/60 vs. 3/28; *P* = 0.675) (S3 Table).

The mrMRF status on pre-CRT MRI showed a statistically significant relevance with respect to tumor relapse, as all tumor relapses developed among positive mrMRF patients (tumor relapse/negative vs. positive mrMRF: n = 0/37 vs. 7/51; *P* = 0.018). The comparison of pre-/post-CRT MRIs identified seven cases of negative mrMRF conversions; therefore, the negative group was re-grouped to consist of 44 patients (no tumor relapses occurred). All tumor relapses (n = 7) occurred among the 44 persistently mrMRF-positive patients (*P* = 0.006) (Table 2).

**Table 2:** Summary of the post-operative tumor relapse rate according to mrMRF status and mrEMVI status assessed on pre-CRT MRI alone (left) and on pre- and post-CRT MRI together (right).

	Pre-CRT MRI		Pre-/post-CRT MRI	
	mrMRF (+)	mrMRF (-)	mrMRF (+)	mrMRF (-)
mrEMVI (+)	22.7% relapse (n = 5/22)	0% relapse (n = 0/5)	27.8% relapse (n = 5/18)	0% relapse (n = 0/3)
mrEMVI (-)	6.9% relapse (n = 2/29)	0% relapse (n = 0/32)	7.7% relapse (n = 2/26)	0% relapse (n = 0/41)

(pre-CRT +: positive, -: negative; pre-/post-CRT +: persistently positive, -: persistently negative and negative conversion)

EMVI, extramural venous invasion; MRF, mesorectal fascia status; CRT, neoadjuvant concurrent chemoradiotherapy.

On pre-CRT MRI, the mrEMVI status was statistically significant with respect to tumor relapse (tumor relapse/negative vs. positive mrEMVI: n = 2/61 vs. 5/27; *P* = 0.026) (Table 2). After CRT, six initially positive mrEMVI patients negatively converted. When we compared the pre-/post-CRT MRI, persistently positive mrEMVI-status patients more frequently developed tumor relapse compared with negative (including negative conversion) mrEMVI-status patients (tumor relapse/negative vs. positive mrEMVI: n = 2/67 vs. 5/21; *P* = 0.008) (Table 2).

**Fig 2:**
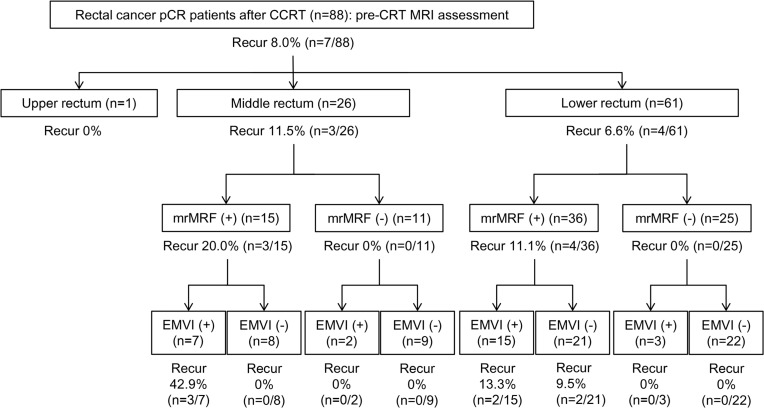
Post-operative tumor relapse rate of pCR rectal cancer patients categorized according to pre-CRT MRI features. The patients are grouped according to the level of the tumor distal margin (upper, middle, or lower rectum) and pre-CRT MRI assessment of mrMRF status and mrEMVI status.

**Fig 3:**
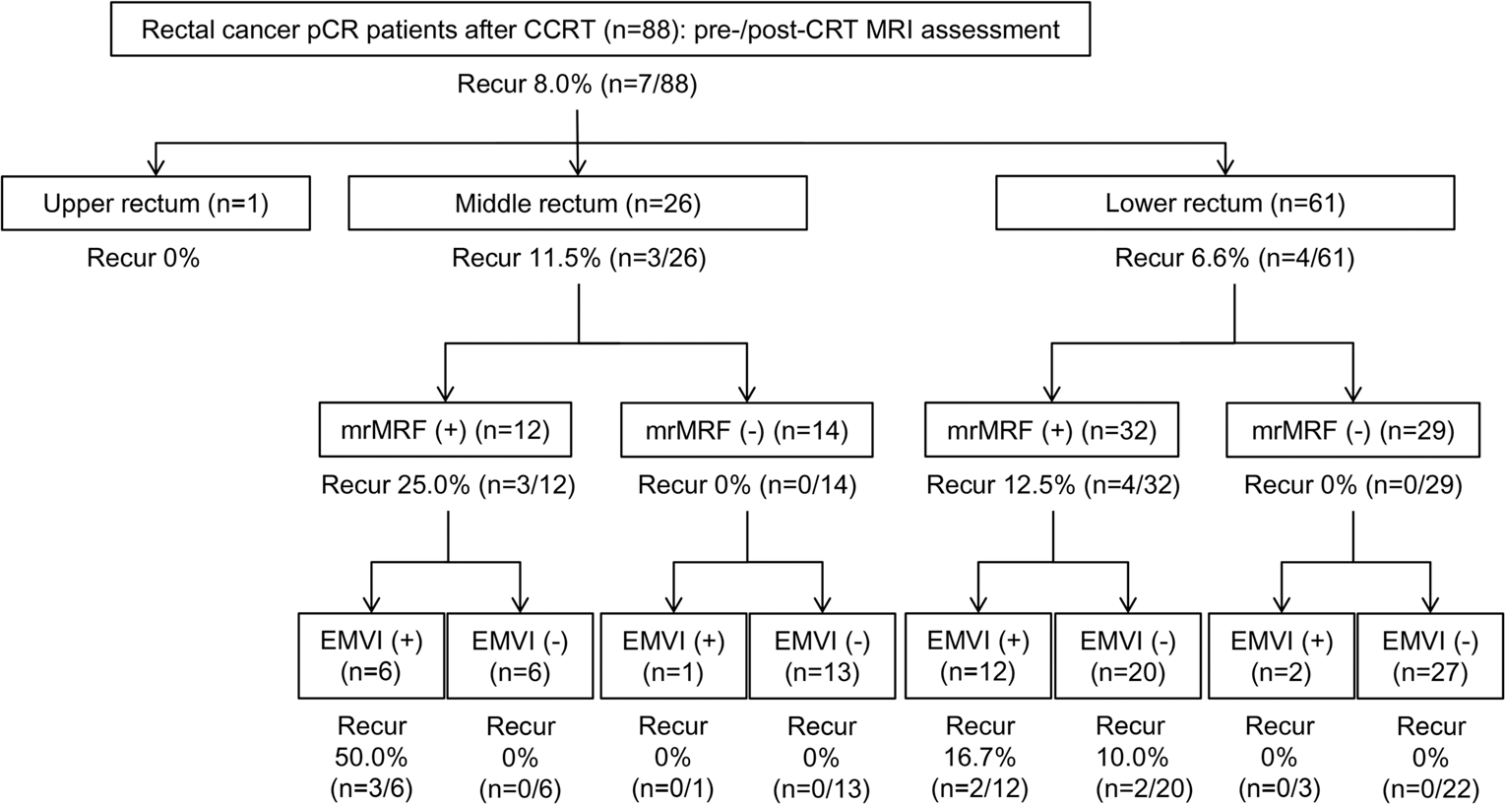
Post-operative tumor relapse rate of pCR rectal cancer patients categorized according to pre/post-CRT MRI features. The patients are grouped according to the level of the tumor distal margin (upper, middle, or lower rectum) along with the assessment results of mrMRF status and mrEMVI status derived from both pre-CRT and post-CRT MRI (+: persistently positive, -: persistently negative and negative conversion).

When the mrMRF and mrEMVI status were considered together (Fig 2) based solely on pre-CRT MRI results, the percentages developing a relapsed tumor were 22.7% (n = 5/22, both factors positive) and 6.9% (n = 2/29, one factor positive), respectively, according to the numbers of positive risk factors. When pre-/post-CRT MRIs were analyzed together (Fig 3), the percentages of tumor relapses increased to 27.8% (n = 5/18, both factors persistently positive) and 7.7% (n = 2/26, one factor persistently positive), according to the number of persistently positive parameters (Table 2).

Based on the pathology report of the surgical specimen, four patients (4.5%, n = 4/88) were identified to carry pathologically confirmed metastatic regional LNs. These four patients were each graded as having negative (n = 2) and positive (n = 2) mrN status on both pre- and post-CRT MRIs. No tumor relapse occurred in these four patients (S2 Table). Pre-CRT mrN status revealed no significant association with tumor relapse (tumor relapse/negative vs. positive mrN: n = 3/50 vs. 4/38; *P* = 0.459). When the pre- and post-CRT MRIs were compared, 14 patients showed persistently positive mrN status (one patient developed a tumor relapse). After CRT, 24 initially positive mrN positive patients experienced negative conversion and 50 patients were persistently negative. Consequently, 74 patients (six patients developed tumor relapse) were mrN-negative (including negative conversion cases) without a significant difference in tumor relapse (*P* = 1.0), (S3 Table).

**Fig 4:**
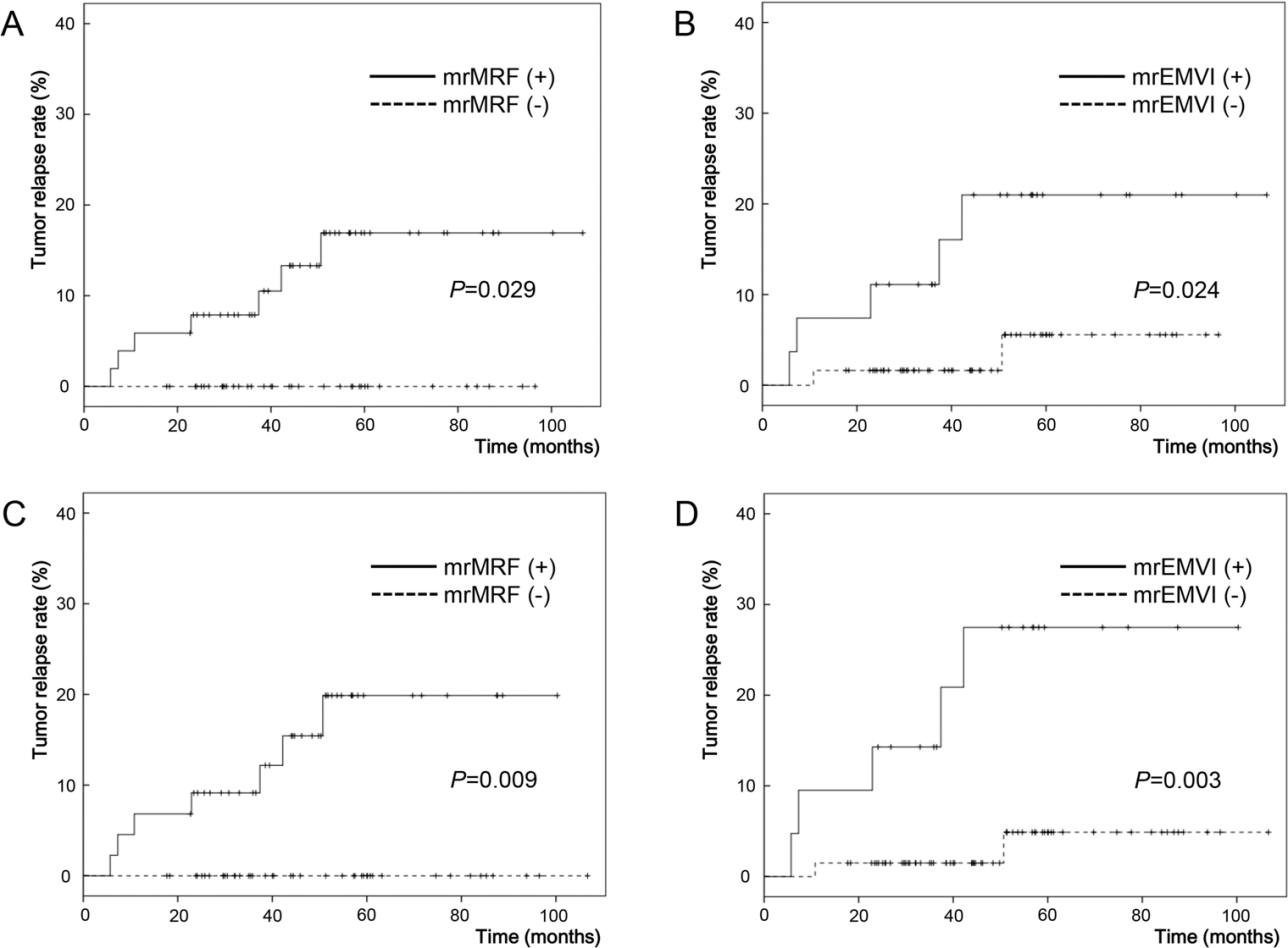
Actuarial probability of the tumor relapse rate. Actuarial probability of the tumor relapse rate according to pre-CRT assessment alone for (A) mrMRF status and (B) mrEMVI status. Actuarial probability when both pre- and post-CRT MRIs are analyzed together for the tumor relapse rate according to (C) mrMRF status and (D) mrEMVI status. (C-D) The positive group consists of patients with persistently positive findings of mrMRF and/or mrEMVI on both pre- and post-CRT MRIs. The negative group includes both persistently negative patients and negatively converted patients.

Kaplan-Meier analysis showed that patients with a positive mrMRF on pre-CRT MRI were at an increased risk of post-operative tumor relapse compared with those with a negative mrMRF (estimated five-year tumor relapse rate: 16.9% vs. 0%, respectively, *P* = 0.029; Fig 4A). Similarly, patients with a positive mrEMVI on pre-CRT MRI were at an increased risk of tumor relapse compared to those with a negative mrEMVI (estimated five-year tumor relapse rate: 21.0% vs. 5.6%, respectively, *P* = 0.024; Fig 4B). No statistically significant difference was observed in overall survival with respect to pre-CRT mrMRF (*P* = 0.165) and mrEMVI (*P* = 0.234) status.

When we compared pre- and post-CRT MRIs, patients with persistently positive mrMRF were at an increased risk of tumor relapse compared with those with a negative mrMRF, including both persistently negative and negative conversion (estimated five-year tumor relapse rate: 19.9% vs. 0%, respectively, *P* = 0.009; Fig 4C). Patients with a persistently positive mrEMVI also showed an increased risk of tumor relapse compared with those with a negative (including both persistently negative and negative conversion) mrEMVI (estimated five-year tumor relapse rate: 27.5% vs. 4.9%, respectively, *P* = 0.003; Fig 4D). Upon comparison of pre- and post-CRT MRIs, the overall survival did not show a statistically significant difference with respect to mrMRF (*P* = 0.090) and mrEMVI (*P* = 0.106) status.

## Discussion

In general, the prognosis for pCR rectal cancer patients is excellent [2, 7]. CRT and total mesorectal excision for locally advanced rectal cancer has an effective role in decreasing the incidence of local recurrence; however, its impact on systemic metastasis is not quite as impressive [1]. Consequently, a post-operative tumor relapse in pCR-achieved patients usually occurs as a distant metastasis. In this study, we observed a 1.1% (n = 1/88) local recurrence rate and 6.8% (n = 6/88) systemic recurrence rate, which together is equivalent to an overall 8.0% (n = 7/88) tumor relapse rate. This is a risk comparable to the 2.6% local recurrence (n = 12/455) and 9.1% (n = 38/419) distant metastasis reported in a meta-analysis study on the long-term outcome of pCR-achieved patients who also had undergone CRT and total mesorectal excision [18]. We hypothesized that a risk stratification of pCR rectal cancer patients to selectively perform intensified treatment in high-risk subpopulations might be possible based on MRI features. To the best of our knowledge, this is the largest study (n = 88) to analyze the MR imaging parameters in association with the outcome of pCR rectal cancer patients who received standard treatment.

The most important finding of this study was that the mrMRF status and mrEMVI status were associated with a risk of post-operative tumor relapse. The mrMRF status corresponds to the circumferential resection margin (CRM) status, which is a strong risk factor of local recurrence and an established prognostic factor [19–21]. Based on rectal cancer study populations who have undergone CRT (but have not been filtered out for pCR cases), Taylor *et al*. demonstrated that CRM involvement detected by either pre-operative MRI or pathological analysis of the surgical specimen is significantly associated with distant metastasis [19]. Our results show that positive mrMRF status serves as a risk factor for tumor relapse in pCR-achieved patients as well.

Positive mrEMVI status was another significant risk factor for post-operative tumor relapse. Theoretically, tumor invaded vessels are a potential source of malignant embolic shower-promoting systemic metastasis, and a positive mrEMVI is an independent risk factor for synchronous metastasis [9]. Similarly, in previous studies (which have not been filtered for pCR patients), EMVI detected either by an MRI or histopathology examination were both tumor relapse risk factors in rectal cancer patients who had undergone CRT [22, 23].

CRT is effective for local tumor control but theoretically has only a limited systemic anti-cancer effect [1]. Therefore, once a patient has been exposed to high-risk factors (such as a positive mrMRF or mrEMVI, in this study), which promote distant metastasis, it seems reasonable to anticipate little preventive effect against systemic metastasis by CRT alone, whether or not the primary cancer reaches pCR state. By definition, pCR patients only carry non-viable devastated remnants of the tumor. As a result, we believe that pathological examination of pCR-achieved patients will probably underestimate the pre-treatment tumor risk factors, even if positive findings were initially present. In contrast, MRI can assess the tumor both before and after CRT. Therefore, at least for the post-operative tumor relapse risk stratification of pCR-achieved patients, we suggest that MRI parameters could be more important than histologic parameters. Moreover, MRI has a practical advantage in that it can be applied to non-surgically treated patients, whose histologic assessment is impossible.

Our data suggest that a single pre-CRT MRI is sufficient to assess mrMRF and mrEMVI status for the prediction of the high-risk group post-operative tumor relapse. However, when the pre- and post-CRT MRIs were analyzed together to identify the patients with persistently positive risk factors, a slightly more efficient risk stratification was possible (Table 2).

Other generally recognized adverse features of rectal cancer treatment include high T and/or N stages, a low rectal location of the lesion, and a high tumor regression grade [5, 24–26]. We analyzed factors such as the pre-treatment CEA level, tumor level, tumor volume, volume reduction rate, mrTRG, mrT, and mrN status. However, under the current context of pCR rectal cancer patients, statistically significant relevance was not observed with respect to the rate of post-operative tumor relapse (S2 and S3 Tables).

In our study population, 4.5% (n = 4/88) of patients were identified to carry pathologically confirmed metastatic LNs, which is a rate similar to one (5%, n = 26/509) that was reported in a large-scale meta-analysis [18]. The ypN stage has been reported to be a predictor for overall tumor relapse [21]; however, none of the four patients in our study developed post-operative tumor relapse. In combination with previous studies [18], this observation supports the notion that less invasive strategies (such as local excision or the ‘wait and see’ approach), which omit histological nodal assessment, should be adopted with caution.

This study has several limitations. Firstly, the events of tumor relapse (n = 7) were insufficient to establish a solid conclusion, and multivariable analysis was not possible. Nevertheless, this is the largest single-center study that has analyzed the MRI features of pCR rectal cancer patients who have undergone standard radical surgery. Secondly, this study is retrospective in nature, and adjuvant chemotherapy was not standardized. Unexpectedly, all the tumor-recurred patients (n = 7) were treated with adjuvant chemotherapy before their tumors relapsed; therefore, the role of adjuvant chemotherapy remains ambiguous. We acknowledge that a randomized prospective study is necessary to validate our findings.

In conclusion, our data suggest that a positive mrMRF and/or positive mrEMVI status are associated with increased risk of tumor relapse in pCR-achieved rectal cancer patients. This can be applied for post-operative risk stratification to individualize therapeutic strategies.

## Supporting Information

S1 Fig MR-based criteria used to classify the rectal cancer level. The Sagittal T2WI of pre-CRT MRI images was used to draw three lines on PACS which each connected the center of symphysis pubis with the 1) sacral promontory (white line), 2) the peritoneal reflection (arrowhead, dotted black line) and 3) the intervertebral junction between the fifth sacral bone and coccyx (black line) to divide the rectum into three compartments (upper rectum: UR, middle rectum: MR and lower rectum: LR). The location of the distal tumor margin assigned the specific rectal compartment of the patient. This example image shows a rectal cancer (arrows) located at the anterior aspect of the rectum, which extends into the middle rectal compartment.
(PDF)

S1 Table Summary of pre-operative MRI findings of the seven pCR-achieved patients who developed post-operative tumor relapse. (DOCX)

S2 Table Summary of the univariate analyses of clinical variables associated with tumor relapse in pCR. (DOCX)

S3 Table Summary of the pre-operative MRI findings of pCR patients according to tumor relapse. (DOCX)
