# Supplementary material for: MRI Risk Stratification for Tumor Relapse in Rectal Cancer Achieving Pathological Complete Remission after Neoadjuvant Chemoradiation Therapy and Curative Resection
Source: PLoS One. 2016 Jan 5;11(1):e0146235. doi: 10.1371/journal.pone.0146235 (PMC4701470; doi:10.1371/journal.pone.0146235)
Supplement: S1 Fig — The Sagittal T2WI of pre-CRT MRI images was used to draw three lines on PACS which each connected the center of symphysis pubis with the 1) sacral promontory (white line), 2) the peritoneal reflection (arrowhead, dotted black line) and 3) the intervertebral junction between the fifth sacral bone and coccyx (black line) to divide the rectum into three compartments (upper rectum: UR, middle rectum: MR and lower rectum: LR). The location of the distal tumor margin assigned the specific rectal compartment of the patient. This example image shows a rectal cancer (arrows) located at the anterior aspect of the rectum, which extends into the middle rectal compartment. (PDF) [file pone.0146235.s001.pdf]

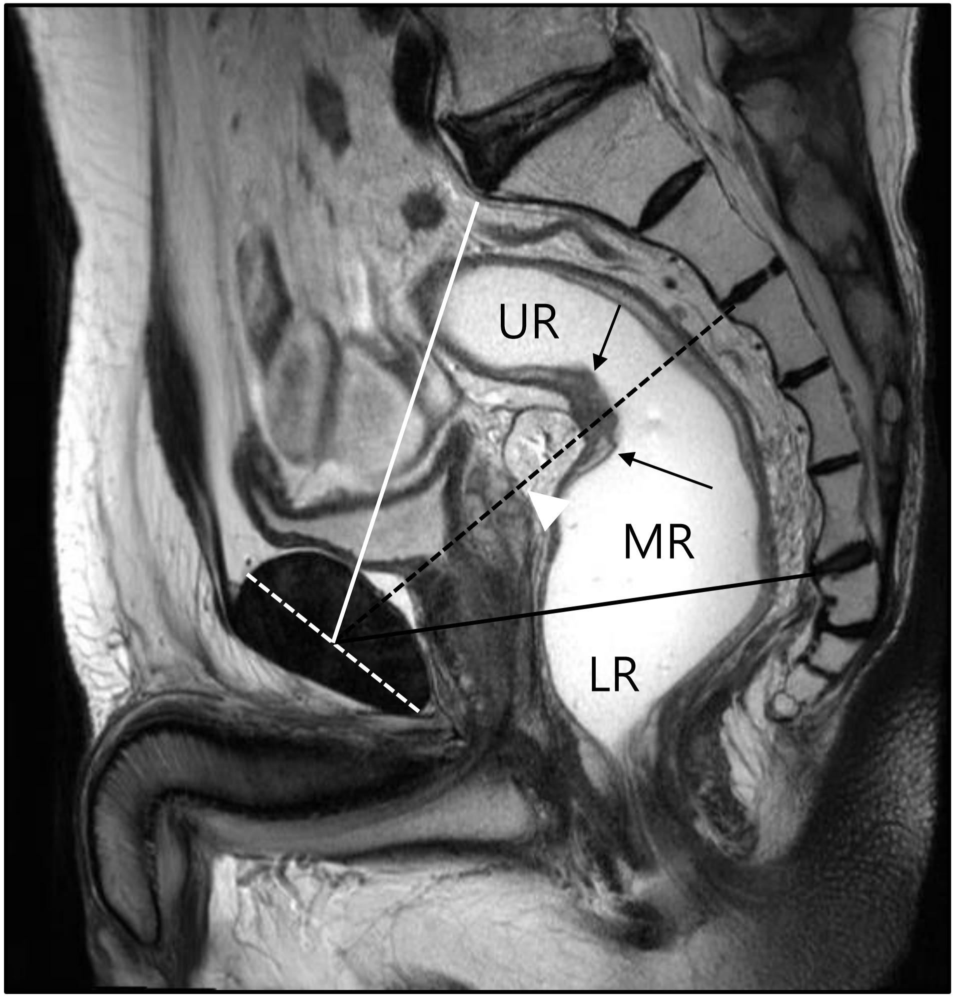

Upper Rectum (UR)
symphysis pubis center ~ sacral promontary

Middle Rectum (MR)
symphysis pubis center ~ peritoneal reflection

Lower Rectum (LR)
symphysis pubis center ~ levator ani muscle posterior insertion
