# Supplementary material for: MRI Risk Stratification for Tumor Relapse in Rectal Cancer Achieving Pathological Complete Remission after Neoadjuvant Chemoradiation Therapy and Curative Resection
Source: PLoS One. 2016 Jan 5;11(1):e0146235. doi: 10.1371/journal.pone.0146235 (PMC4701470; doi:10.1371/journal.pone.0146235)
Supplement: S1 Table — (DOCX) [file pone.0146235.s002.docx]

S1 Table. Summary of pre-operative MRI findings of the seven pCR-achieved patients who developed post-operative tumor relapse.

|  | Sex/age | Site of relapse | Initial tumor level | Relapse-free period (months) | mrT stage | | mrCRM | | Volume | | | mrTRG | mrEMVI | | Regional N stage | | Adjuvant chemotherapy before tumor relapse | Final outcome |
| --- | --- | --- | --- | --- | --- | --- | --- | --- | --- | --- | --- | --- | --- | --- | --- | --- | --- | --- |
|  |  |  |  |  | Pre-CRT | Post-CRT | Pre-CRT | Post-CRT | Pre-CRT | Post-CRT | Reduction rate (%) |  | Pre-CRT | Post-CRT | Pre-CRT | Post-CRT |  |  |
| 1 | M/62 | Liver | Middle rectum | 5.7 | mrT3_≥5mm_ | mrT3_<5mm_ | + | + | 33.1 | 5.2 | 84.3 | 3 | + | + | + | - | FOLFOX | No evidence of disease |
| 2 | M/59 | Para-aortic LNs | Middle rectum | 7.3 | mrT3_≥5mm_ | mrT3_≥5mm_ | + | + | 24.8 | 8.4 | 66.1 | 3 | + | + | + | + | FOLFOX | death |
| 3 | F/71 | Lung | Lower rectum | 10.8 | mrT3_<5mm_ | mrT3_<5mm_ | + | + | 5.6 | 1.0 | 82.1 | 2 | - | - | - | - | FOLFOX | death |
| 4 | F/51 | Lung | Lower rectum | 22.9 | mrT3_<5mm_ | mrT3_<5mm_ | + | + | 29.1 | 14.3 | 50.9 | 3 | + | + | - | - | capecitabine | No evidence of disease |
| 5 | M/70 | Lung | Lower rectum | 37.4 | mrT3_≥5mm_ | mrT3_≥5mm_ | + | + | 81.6 | 32.8 | 59.8 | 3 | + | + | + | - | FOLFOX | death |
| 6 | M/51 | Liver | Middle rectum | 42.2 | mrT3_≥5mm_ | mrT3_≥5mm_ | + | + | 25.9 | 9.7 | 62.4 | 3 | + | + | + | - | FOLFOX | No evidence of disease |
| 7 | F/62 | Anastomosis | Lower rectum | 50.7 | mrT3_<5mm_ | mrT3_<5mm_ | + | + | 10.4 | 1.3 | 87.5 | 2 | - | - | - | - | FOLFOX | No evidence of disease |

MRF, mesorectal fascia status; EMVI, extramural venous invasion; TRG, tumor regression grade; CRT, neoadjuvant concurrent chemoradiotherapy
