# Supplementary material for: MRI Risk Stratification for Tumor Relapse in Rectal Cancer Achieving Pathological Complete Remission after Neoadjuvant Chemoradiation Therapy and Curative Resection
Source: PLoS One. 2016 Jan 5;11(1):e0146235. doi: 10.1371/journal.pone.0146235 (PMC4701470; doi:10.1371/journal.pone.0146235)
Supplement: S2 Table — (DOCX) [file pone.0146235.s003.docx]

S2 Table. Summary of the univariate analyses of clinical variables associated with tumor relapse in pCR.

| Clinical Variables | | Tumor relapse | | *P* |
| --- | --- | --- | --- | --- |
|  |  | Yes (n=7) | No (n=81) |  |
| Age | ≤60 | 3 | 45 | 0.697 |
|  | >60 | 4 | 36 |  |
| Sex | Male | 4 | 52 | 0.702 |
|  | Female | 3 | 29 |  |
| Pre-CRT CEA | Mean±S.D. (ng/mL) | 16.0±6.1 | 4.0±6.0 | 0.471 |
| Histologic grade | Well-differentiated | 2 | 19 | 0.073 |
|  | Moderately differentiated | 3 | 58 |  |
|  | Poorly differentiated | 1 | 2 |  |
|  | Mucinous | 1 | 2 |  |
| Pathologic LN metastasis | (-) | 0 | 84 | 1.0 |
|  | (+) | 0 | 4 |  |
| CRT protocol | 5-fluorouracil+leucovorin | 3 | 57 | 0.131 |
|  | Capecitabine | 0 | 6 |  |
|  | Irinotecan/TS-1 | 4 | 18 |  |
| Adjuvant chemotherapy | None | 0 | 23 | 0.184 |
|  | FOLFOX protocol | 6 | 53 |  |
|  | Capecitabine | 1 | 4 |  |
|  | FOLFIRI protocol+Avastin | 0 | 1 |  |

LN, lymph node; CRT, neoadjuvant concurrent chemoradiotherapy.
