# Supplementary material for: MRI Risk Stratification for Tumor Relapse in Rectal Cancer Achieving Pathological Complete Remission after Neoadjuvant Chemoradiation Therapy and Curative Resection
Source: PLoS One. 2016 Jan 5;11(1):e0146235. doi: 10.1371/journal.pone.0146235 (PMC4701470; doi:10.1371/journal.pone.0146235)
Supplement: S3 Table — (DOCX) [file pone.0146235.s004.docx]

S3 Table. Summary of the pre-operative MRI findings of pCR patients according to tumor relapse.

| MRI variables | | | Tumor relapse | | *P* |
| --- | --- | --- | --- | --- | --- |
|  |  |  | Yes (n=7) | No (n=81) |  |
| Tumor level | Pre-CRT | Upper | 0 | 1 | 0.468 |
|  |  | Middle | 3 | 23 |  |
|  |  | Lower (including anal canal) | 4 | 57 |  |
| mrT stage | Pre-CRT | mrT3_<5mm_ | 3 | 43 | 0.705 |
|  |  | mrT3_≥5mm_ | 4 | 38 |  |
|  | Post-CRT | mrT3_<5mm_ | 4 | 56 | 0.675 |
|  |  | mrT3_≥5mm_ | 3 | 25 |  |
| mrN stage | Pre-CRT | - | 3 | 47 | 0.459 |
|  |  | + | 4 | 34 |  |
|  | Post-CRT | - | 6 | 68 | 1.0 |
|  |  | + | 1 | 13 |  |
| mrTRG |  | mrTRG_1/2_ | 2 | 24 | 1.0 |
|  |  | mrTRG_3/4/5_ | 5 | 57 |  |
| MR volume | Pre-CRT (cm^3^) | Mean±S.D. | 30.1±24.9 | 23.9±24.4 | 0.516 |
|  | Post-CRT (cm^3^) | Mean±S.D. | 10.3±11.1 | 7.4±8.9 | 0.421 |
|  | Reduction rate (%) | Mean±S.D. | 70.3±14.0 | 70.7±19.8 | 0.962 |
| mrMRF | Pre-CRT | - | 0 | 37 | 0.018 |
|  |  | + | 7 | 44 |  |
|  | Post-CRT | - | 0 | 44 | 0.006 |
|  |  | + | 7 | 37 |  |
| mrEMVI | Pre-CRT | - | 2 | 59 | 0.026 |
|  |  | + | 5 | 22 |  |
|  | Post-CRT | - | 2 | 65 | 0.008 |
|  |  | + | 5 | 16 |  |

LN, lymph node; EMVI, extramural venous invasion; TRG, tumor regression grade; N, nodal status; pCR, pathological complete remission; CRT, neoadjuvant concurrent chemoradiotherapy.
